# Supplementary material for: Heme-deficient metabolism and impaired cellular differentiation as an evolutionary trade-off for human infectivity in Trypanosoma brucei gambiense
Source: Nat Commun. 2022 Nov 18;13:7075. doi: 10.1038/s41467-022-34501-4 (PMC9674590; doi:10.1038/s41467-022-34501-4)
Supplement: Supplementary file 1 — Supplementary information [file 41467_2022_34501_MOESM1_ESM.pdf]

Supplementary information: **Heme-deficient metabolism and impaired cellular differentiation as an evolutionary trade-off for human infectivity in *Trypanosoma brucei gambiense***

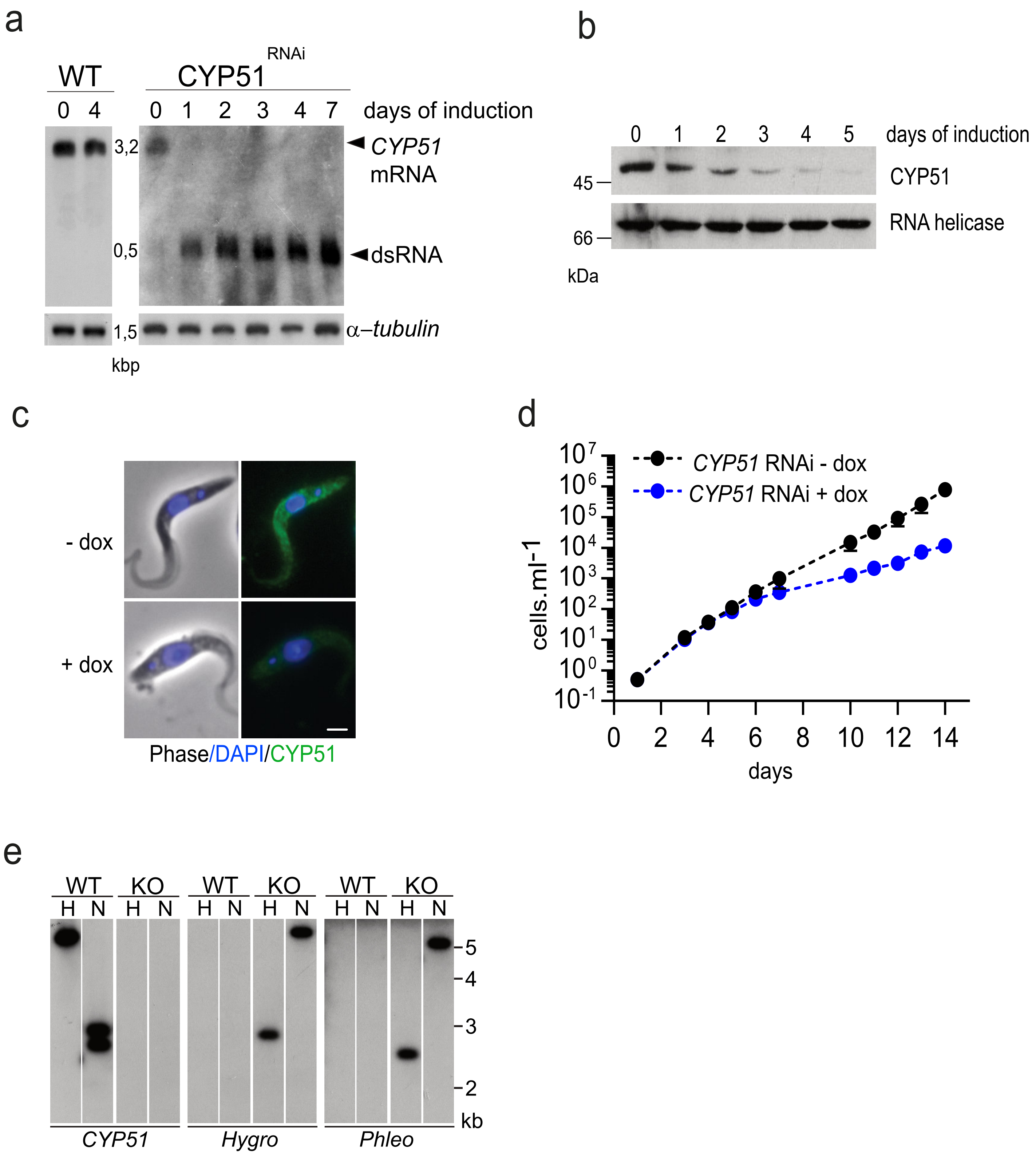

Suppl. Figure 1. Characterization of *TbCYP51* RNAi and knock-out in the procyclic stage
(**a**) Total RNA from WT *T. b. brucei* and *CYP51* RNAi cell line was analyzed by Northern blot hybridized with the *CYP51* probe. α-tubulin was used as a loading control. Arrowheads indicate *CYP51* mRNA and doxycyclin-induced dsRNA. (**b**) Total protein extracts from the *CYP51* RNAi cell line were analyzed by Western blot with anti-CYP51 and anti-RNA helicase as a loading control. (**c**) *TbCYP51* RNAi cell line was labeled by indirect immunofluorescence with anti-CYP51 antibody. DNA was stained with DAPI. Scale bar, 3 μm. (**d**) Cumulative growth curve comparing the non-induced (-dox) and induced (+dox) *CYP51* RNAi cells. (**e**) Southern blot analysis of total DNA isolated from WT *T. b. brucei* and CYP51 KO digested with restriction enzyme *Hind*III (H) or *Nsi*I (N). The membrane was hybridized with a radiolabeled probe from CYP51, hygromycin- or phleomycin-resistance genes. Source data are provided as a Source Data file.

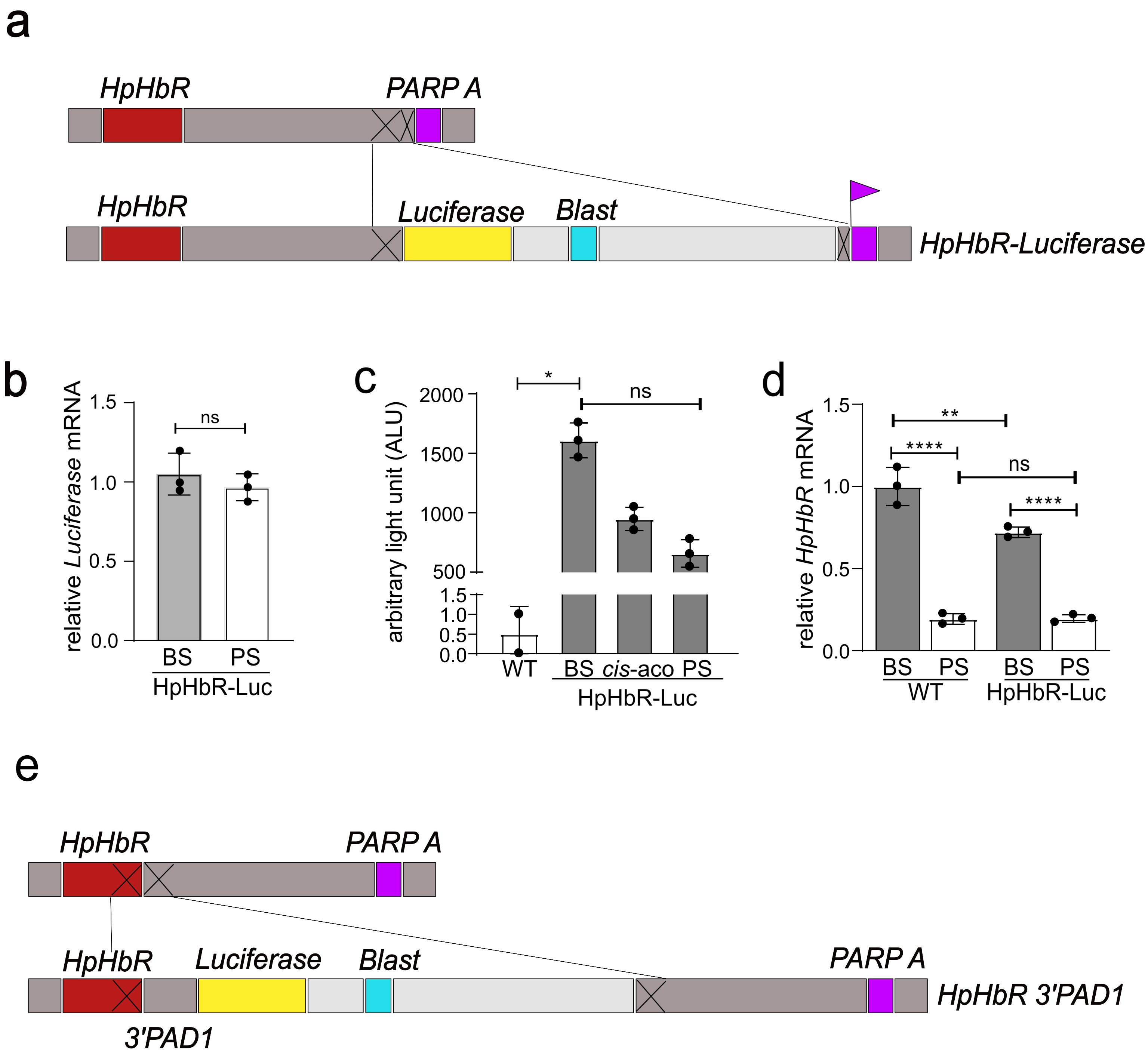

Suppl. Figure 2. Artificial expression of HpHbR in stumpy form does not interfere with life cycle progression
(**a**) Scheme showing the generation of *HpHbR-Luc* construct. *T. b. brucei* wild type (90-13) was engineered to express *Luciferase* downstream *HpHbR* at the end of the polycistronic transcription unit. (**b**) *In vitro* differentiation was triggered by 3 mM cis-aconitate (*cis-aco*). The Luciferase mRNA levels were evaluated 2 hours after *cis-aco* addition *via* RT-qPCR with H2B gene used as an internal control. (n=3) (**c**) The luciferase activity was measured in HpHbR-Luc cell line in its bloodstream stage (BS; n=3), 2 h after differentiation with *cis*-aconitate (*cis*-aco; n=3), and in the procyclic stage (PS; n=3), as well as in wild types (WT; n=2), which were used as a negative control. (**d**) RT-qPCR assessed the expression of HpHbR in WT and HpHbR-Luc cells. RNA was extracted from the bloodstream (BS) and procyclic stages (PS) of individual cell lines and used for cDNA production. (n=3). n represents biologically independent experiments; Error bars indicate ±SD. (**e**) Scheme showing the generation of HpHbR *3'PAD1* construct. *T. b. brucei* wild type (90-13) *was* engineered to express *HpHbR* ORF under the control of stumpy specific *PAD1* 3'UTR. Source data are provided as a Source Data file.

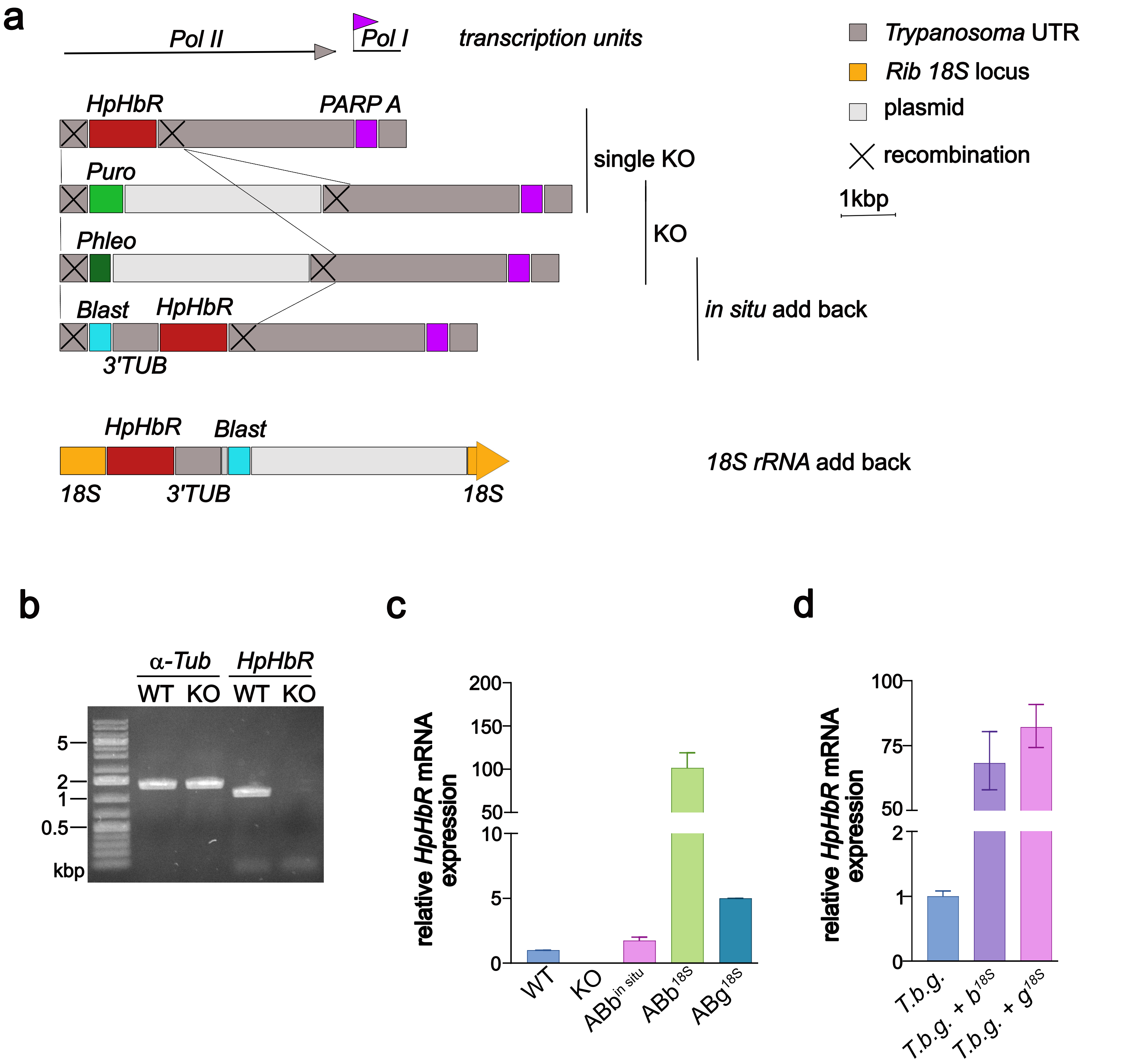

Suppl. Figure 3. Generation of HpHbR KO and addback cell lines

(**a**) Steps of HpHbR invalidation and addbacks expression in the 90:13 *T. b. brucei* cell line. The *TbHpHbR* locus is located at the end of a Pol II transcribed unit. The first allele was replaced with pPuroKO construct and the second allele with pPhleoKO construct. ABb$^{in\ situ}$ cell line was obtained by targeting the phleomycin allele with a PCR product containing *Tb. brucei HpHbR* ORF and the Blasticidin resistance gene. ABb^{18S} cell line was obtained by targeting the 18S rRNA locus with the construct containing *T. b. brucei HpHbR* ORF and the Blasticidin resistance gene. All constructs are drawn at scale. For clarity, *Trypanosoma* UTRs contained in the plasmids have not been depicted. (**b**) Verification of HpHbR KO by PCR. Relative expression of HpHbR in individual cell lines (**c**) *T.b. brucei* AnTat1.1 90-13 (WT), HpHbR KO (KO), addbacks ABb$^{in\ situ}$ and ABb^{18S} (**d**) *T.b. gambiense* LiTat 1.3 (*T. b. gambiense*), *T.b. gambiense* strain overexpressing different variants of HpHbR from 18S rRNA locus (*T.b. brucei* HpHbR = *T.b.g.*+ b^{18S,} *T.b. gambiense* HpHbR = *T.b.g.*+ g^{18S}). Data are derived from 3 technical replicates. Error bars indicate ±SD. Source data are provided as a Source Data file.

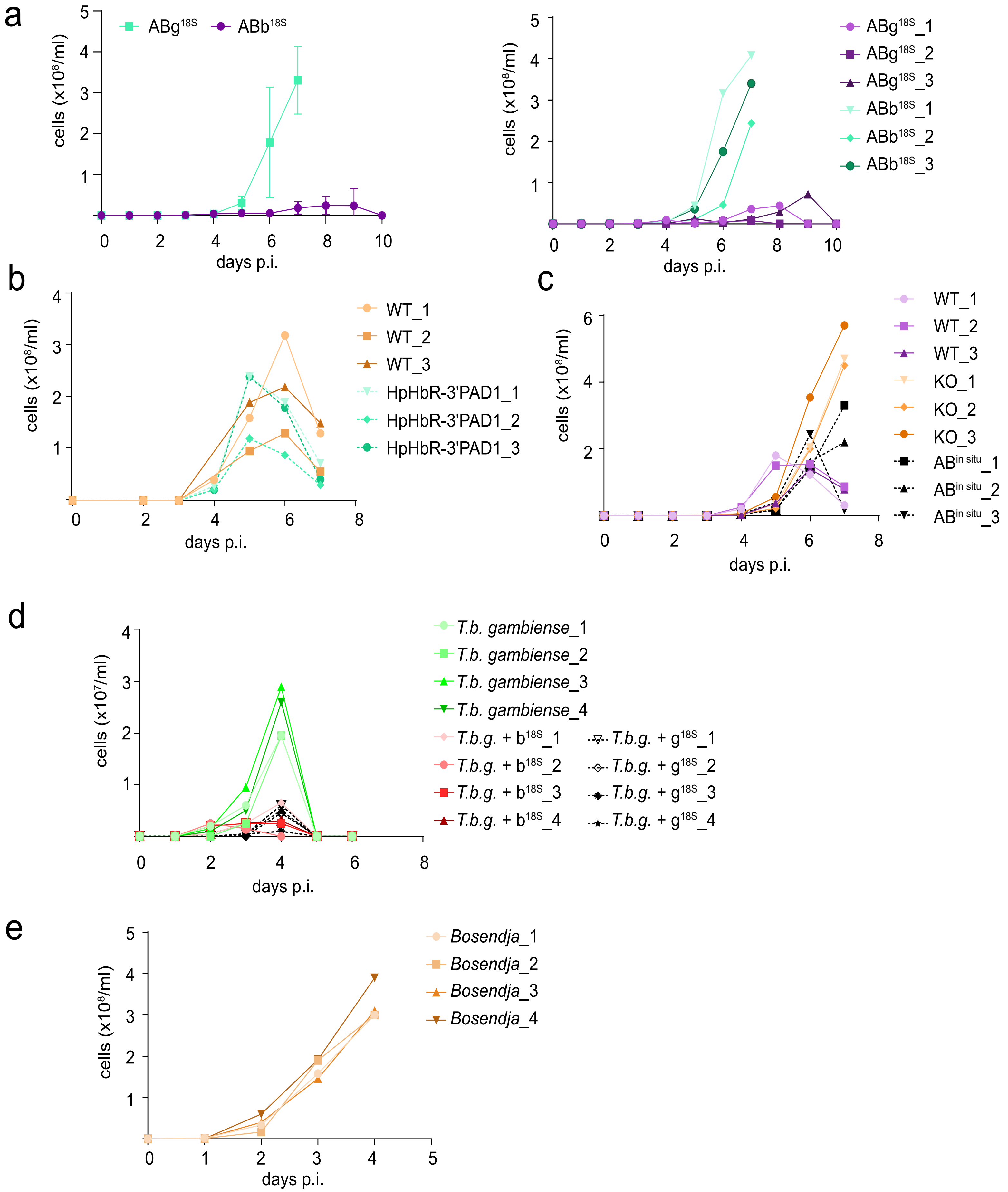

Suppl. Figure 4. An individual *in vivo* parasitemia of strains from the study

(**a**) *In vivo* infectivity of *T.b. brucei* addback cell lines ABb^{18S} (green line) and ABg^{18S} (purple line) was evaluated by infecting mice with 1 x 10^4 cells (n=3). The parasitemia was counted daily. Error bars indicate ±SD. The left panel shows a representative graph out of 3 biological replicates. The same experiment is shown for each infected animal, right panel. Representative *in vivo* parasitemia of different strains from the study is shown for each infected animal (**b**) extended data to Fig. 3B (n=3) (**c**) extended data to Fig. 4E (n=3) (**d**) extended data to Fig. 5A (n=4) (**e**) extended data to Fig. 5E (n=4). Source data are provided as a Source Data file.

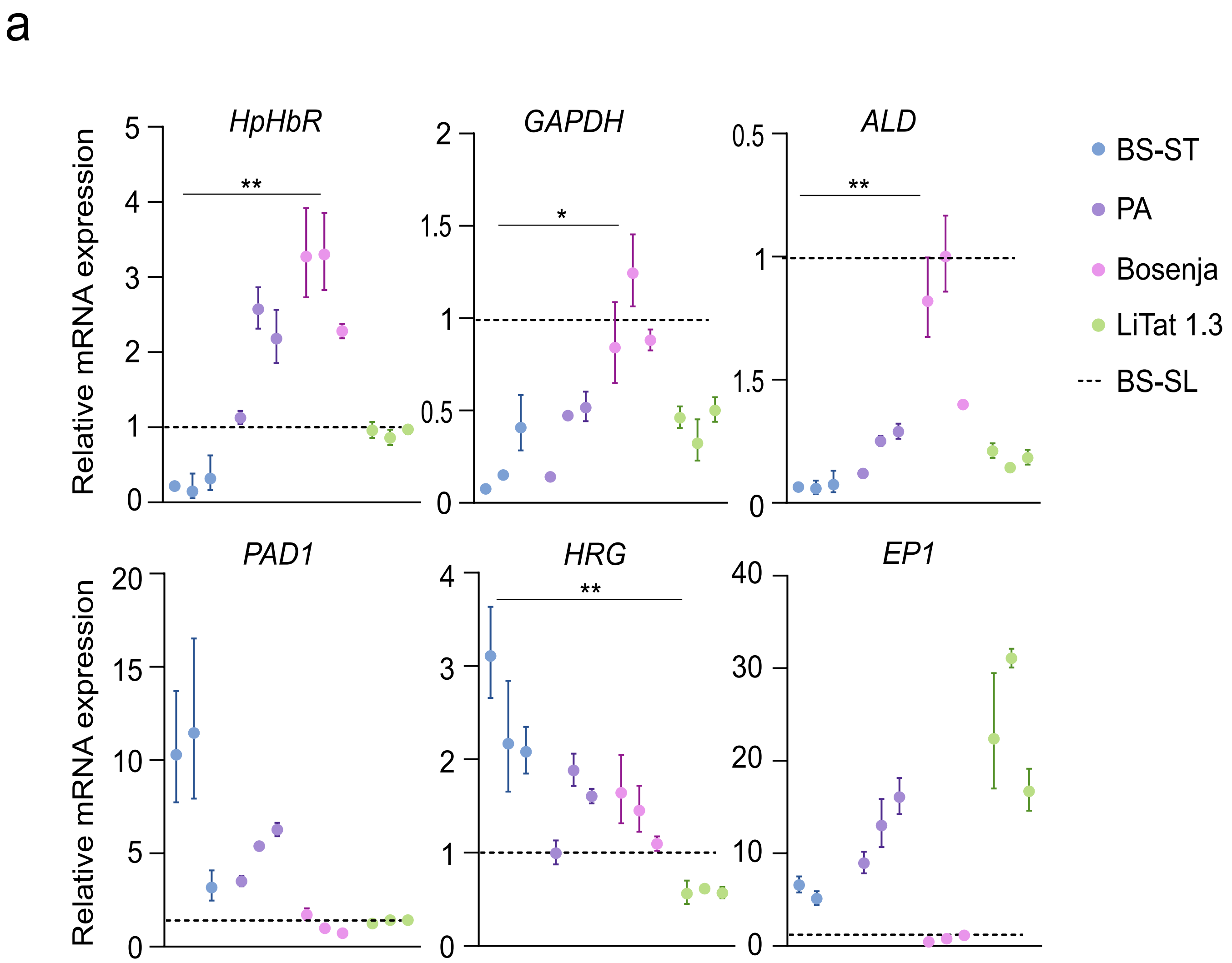

Suppl. Figure 5. Evaluation of stumpy markers in different *T.b. gambiense* strains

(**a**) RT-qRT PCR analysis of genes downregulated (*HpHbR* Tb427.06.440, *GAPDH* Tb927.6.4280, *ALD* Tb427.10.5620; upper panel) and upregulated (*PAD1* Tb927.7.5930, *HRG* Tb927.8.6010, *EP1* Tb927.10.10260; lower panel) in BS-ST cells. RNA was isolated from *ex vivo* cells (the day of harvesting from Balb/c mouse is stated in the legend). Different *T.b. gambiense* strains (LiTat 1.3, Bosendja and PA) were compared to BF-ST (blue dots) and BF-SL controls (dotted line), respectively (n=3, LiTat 1.3 n=2). Dunn's multiple comparison test *p<0.01; **p<0.001. Error bars indicate ±SD. Source data are provided as a Source Data file.

Uncropped scans and images

Suppl. Fig. 1a:

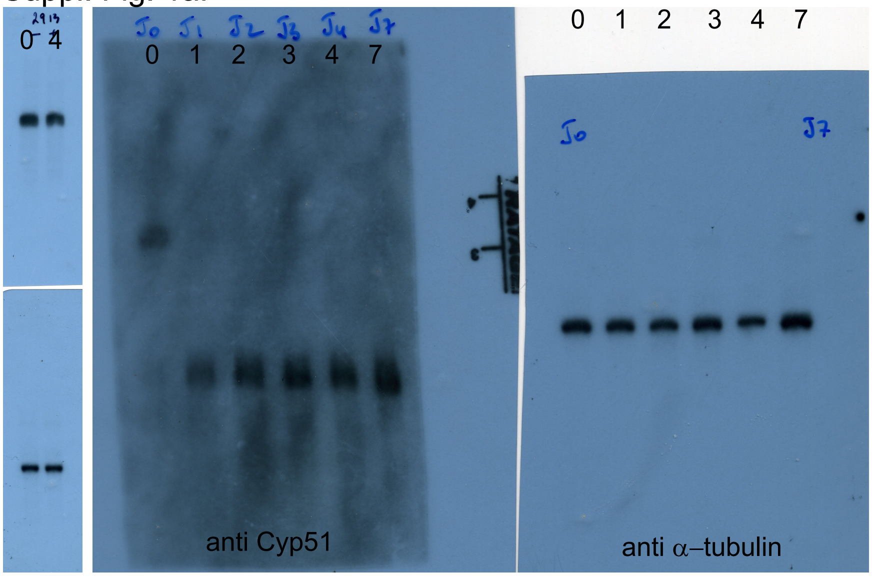

WT

Cyp51 RNAi

Suppl. Fig. 1b:

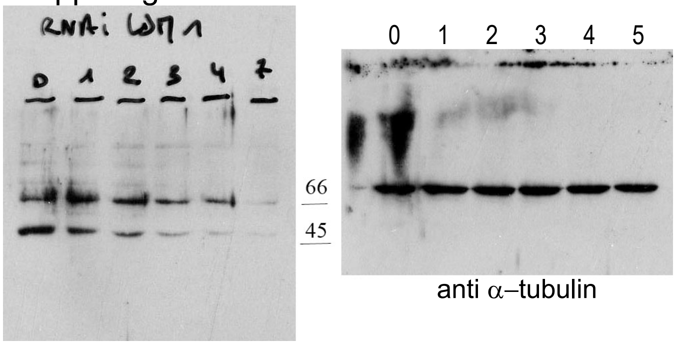

anti Cyp51

Suppl. Fig. 1e:

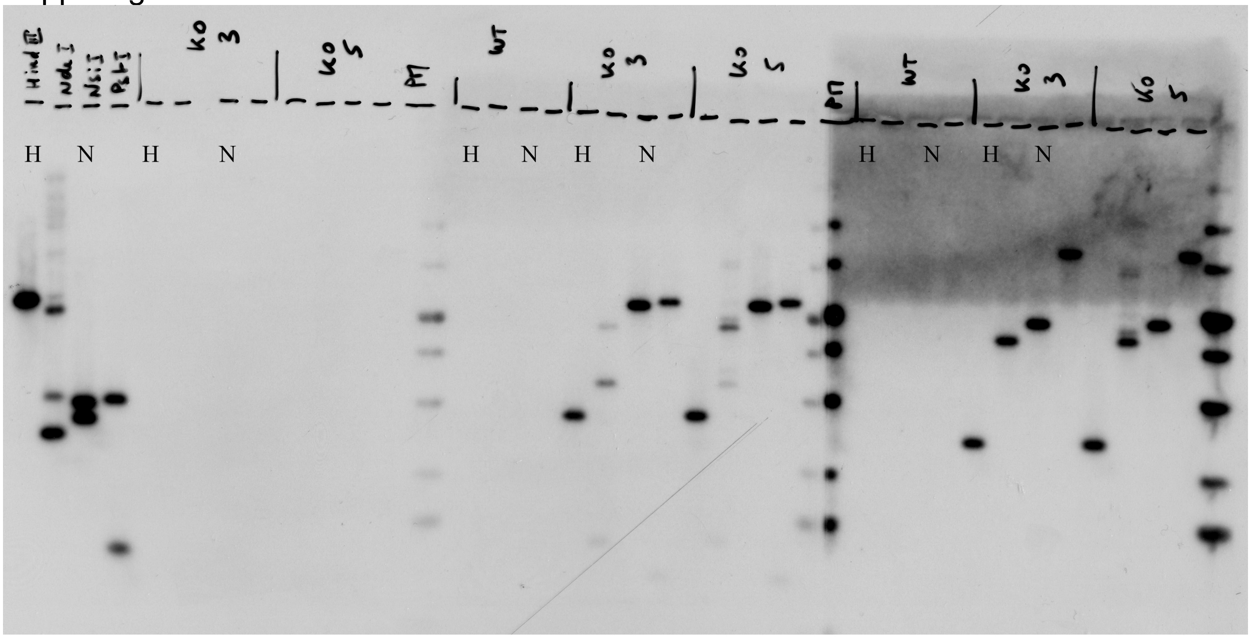

CYP51 HYGRO PHLEO

Suppl. Fig. 3b:

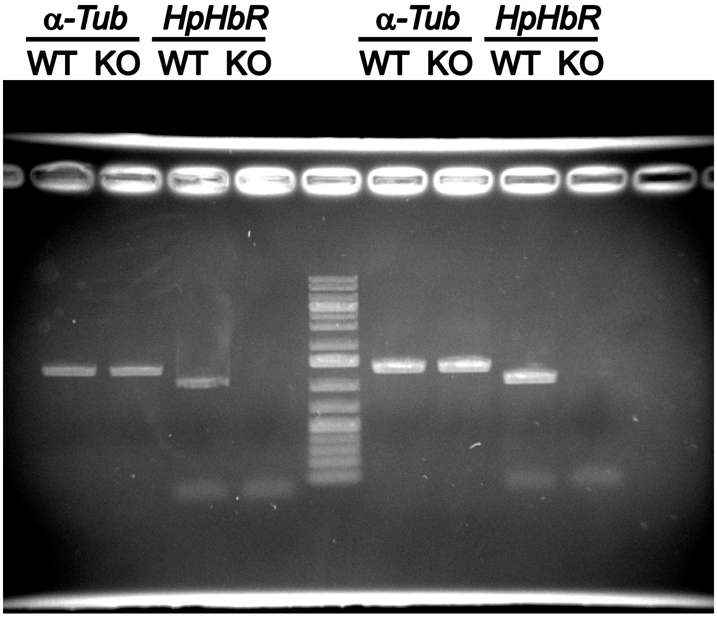

repeat 1 repeat 2- used

Supplementary Table 1

Primers used in this study:

gene	accession #	FW primer	RV primer
HRG	Tb927.8.6010	TTTCGTACCTTGTGGGAGCC	ATAACGTGCATGGCGATTGC
https://tritrypdb.org/tritrypdb/app/record/gene/Tb927.8.6010			
GAPDH	Tb927.6.4280	ATCATCCCAAGCACCACTGG	CGCGTCGCAATGAAGGTAAG
https://tritrypdb.org/tritrypdb/app/record/gene/Tb927.6.4280			
EP1	Tb927.10.10260	CGTTCCCTTTATCTGCTCGC	CAACGAGAGCAGCAGCCG
https://tritrypdb.org/tritrypdb/app/record/gene/Tb927.10.10260			
ALD	Tb427.10.5620	TCATGCTGGAATGCGAAGGT	TGTTTCGCCGGTCTTAGCTT
https://tritrypdb.org/tritrypdb/app/record/gene/Tb427.10.5620			
HpHbR	Tb427.06.440	GAGGGGTCTTGTTGTGGAAA	ACTGGCATAACTGCGGGAAAC
https://tritrypdb.org/tritrypdb/app/record/gene/Tb427.06.440			
PAD1	Tb927.7.5930	TACACATATTGTACGAGCAG	TCATAAACGAGAGCTAAGTG
https://tritrypdb.org/tritrypdb/app/record/gene/Tb927.7.5930			
LUC	LOC116160065	GCAAAACGCTTCCATCTTCC	TCCACAACCTTCGCTTCAAA
https://pubchem.ncbi.nlm.nih.gov/gene/LOC116160065			
C1	Tb927.10.12970	TTGTGACGACGAGAGCAAAC	GAAGTGGTTGAACGCCAAAT
https://tritrypdb.org/tritrypdb/app/record/gene/Tb927.10.12970			
