# Supplementary material for: Heme-deficient metabolism and impaired cellular differentiation as an evolutionary trade-off for human infectivity in Trypanosoma brucei gambiense
Source: Nat Commun. 2022 Nov 18;13:7075. doi: 10.1038/s41467-022-34501-4 (PMC9674590; doi:10.1038/s41467-022-34501-4)
Supplement: Supplementary file 3 — Reporting Summary [file 41467_2022_34501_MOESM3_ESM.pdf]

Corresponding author(s): Eva Horáková
Last updated by author(s): Sep 24, 2022

Reporting Summary

Nature Research wishes to improve the reproducibility of the work that we publish. This form provides structure for consistency and transparency in reporting. For further information on Nature Research policies, see our Editorial Policies and the Editorial Policy Checklist.

Statistics

For all statistical analyses, confirm that the following items are present in the figure legend, table legend, main text, or Methods section.

n/a	Confirmed	
☐	☒	The exact sample size (n) for each experimental group/condition, given as a discrete number and unit of measurement
☐	☒	A statement on whether measurements were taken from distinct samples or whether the same sample was measured repeatedly
☐	☒	The statistical test(s) used AND whether they are one- or two-sided<br>*Only common tests should be described solely by name; describe more complex techniques in the Methods section.*
☒	☐	A description of all covariates tested
☒	☐	A description of any assumptions or corrections, such as tests of normality and adjustment for multiple comparisons
☐	☒	A full description of the statistical parameters including central tendency (e.g. means) or other basic estimates (e.g. regression coefficient) AND variation (e.g. standard deviation) or associated estimates of uncertainty (e.g. confidence intervals)
☐	☒	For null hypothesis testing, the test statistic (e.g. F, t, r) with confidence intervals, effect sizes, degrees of freedom and P value noted<br>*Give P values as exact values whenever suitable.*
☒	☐	For Bayesian analysis, information on the choice of priors and Markov chain Monte Carlo settings
☒	☐	For hierarchical and complex designs, identification of the appropriate level for tests and full reporting of outcomes
☒	☐	Estimates of effect sizes (e.g. Cohen's d, Pearson's r), indicating how they were calculated

Our web collection on statistics for biologists contains articles on many of the points above.

Software and code

Policy information about availability of computer code

Data collection	Excel 16.0.15028.20160, Olympus cellSens Standard Imaging Software v. 1.15, Oroboros Datlab version 7.4.0.4, Biorad Image Lab 6.0.1
Data analysis	Fiji v. 2.3.1., Image J v. 1.8.0., Adobe Photoshop CC 2019, GraphPad Prism 8

For manuscripts utilizing custom algorithms or software that are central to the research but not yet described in published literature, software must be made available to editors and reviewers. We strongly encourage code deposition in a community repository (e.g. GitHub). See the Nature Research guidelines for submitting code & software for further information.

Data

Policy information about availability of data

All manuscripts must include a data availability statement. This statement should provide the following information, where applicable:

- Accession codes, unique identifiers, or web links for publicly available datasets
- A list of figures that have associated raw data
- A description of any restrictions on data availability

The data generated during and/or analysed during the current study are available from the corresponding author on request.

Field-specific reporting

Please select the one below that is the best fit for your research. If you are not sure, read the appropriate sections before making your selection.

☒ Life sciences ☐ Behavioural & social sciences ☐ Ecological, evolutionary & environmental sciences

For a reference copy of the document with all sections, see nature.com/documents/nr-reporting-summary-flat.pdf

Life sciences study design

All studies must disclose on these points even when the disclosure is negative.

Sample size	The sample size for the infection in the animal model (BALB/c mice) was n=5 per experiment, the number of flies dissected was 37, 38, and 40, respectively. No sample size calculation was performed here; we set the number of animals from our previous experimental experience with the models to be minimal and still informative enough.
Data exclusions	The mice which remained uninfected were excluded.
Replication	Each experiment had at least three biological replicates. The replicates were all successful.
Randomization	The experimental organisms where allocated into experimental groups based on the individual cell line used. All experiments included control group with the same number of cells.
Blinding	The investigators were not blinded to the conditions measured. There were multiple people involved in the analysis of the data. Each group verified the data independently. We agree that blinding would be even more appropriate, but unfortunately not routinely performed at that time by us.

Reporting for specific materials, systems and methods

We require information from authors about some types of materials, experimental systems and methods used in many studies. Here, indicate whether each material, system or method listed is relevant to your study. If you are not sure if a list item applies to your research, read the appropriate section before selecting a response.

Materials & experimental systems

n/a	Involved in the study
☐	☒ Antibodies
☐	☒ Eukaryotic cell lines
☒	☐ Palaeontology and archaeology
☐	☒ Animals and other organisms
☒	☐ Human research participants
☒	☐ Clinical data
☒	☐ Dual use research of concern

Methods

n/a	Involved in the study
☒	☐ ChIP-seq
☒	☐ Flow cytometry
☒	☐ MRI-based neuroimaging

Antibodies

Antibodies used	V5 Tag Mouse Monoclonal Antibody (Thermo Fischer Scientific product #37-7500, clone 2F11F7), Monoclonal Anti-α-Tubulin antibody produced in mouse (Sigma product #T5168, clone B-5-1-2), PAD1 (custom made kind gift from Keith Matthews, University of Edinburgh), CYP51 (custom made, source Benoit Vanhollebeke), Enolase (custom made kind gift from Paul Michels, University of Edinburgh), secondary Alexa Fluor-488 anti-rabbit IgG antibody (Thermo Fischer Scientific #A-11034), Alexa Fluor-555 anti-rabbit IgG antibody (Thermo Fischer Scientific #A-21428) and Alexa Fluor-488 anti mouse IgG antibody (Thermo Fischer Scientific #A-11001).
Validation	V5 Tag Mouse Monoclonal Antibody- used in numerous studies (e.g. Liu, Dingxie et al. "Genetic alterations in the phosphoinositide 3-kinase/Akt signaling pathway confer sensitivity of thyroid cancer cells to therapeutic targeting of Akt and mammalian target of rapamycin." Cancer research vol. 69,18 (2009): 7311-9.) Monoclonal Anti-α-Tubulin antibody produced in mouse - used in numerous studies (e.g. Hirst, William G et al. "Differences in Intrinsic Tubulin Dynamic Properties Contribute to Spindle Length Control in Xenopus Species." Current biology : CB vol. 30,11 (2020): 2184-2190.e5.). We tested the antibody against transgenic Trypanosoma brucei, using it several dilutions (1:1000; 1:5000; 1:10 000). 1:1000 dilutions resulted in optimal and highly specific signal recognizing the V5 tag.<br>Monoclonal Anti-α-Tubulin antibody produced in mouse (Sigma product #T5168, clone B-5-1-2)- used in numerous studies (e.g. Piperno, G., et al., J. Cell Biol., 104, 289, 1987; LeDizet, M., and Piperno, G., Meth. Enzymol., 196, 264, 1991; LeDizet, M., and Piperno, G., Proc. Natl. Acad. Sci. USA, 84, 5720, 1987; Bulinski, J., et al., J. Cell Biol., 106, 1213, 1988; LeDizet, M., and Piperno, G., J. Cell Biol., 104, 13, 1986.) We tested the antibody against Trypanosoma brucei using several dilutions (1:1000; 1:5000; 1:10 000). 1:10 000 dilutions resulted in optimal and highly specific signal recognizing α-Tubulin.<br>Custom-made antibodies were used according to the published protocols in T. brucei. (PAD1 Ab-Dean S, Marchetti R, Kirk K, Matthews KR. A surface transporter family conveys the trypanosome differentiation signal. Nature. 2009;459(7244):213–7. 10.1038/

nature07997; enolase Ab- Hannaert V, Albert MA, Rigden DJ, da Silva Giotto MT, Thiemann O, et al. (2003) Kinetic characterization, structure modelling studies and crystallization of Trypanosoma brucei enolase. Eur J Biochem 270: 3205–3213.).
All used fluorochrome (Alexa 488, Alexa 555) coated secondary antibodies were used in 1: 1000 dilutions; conditions that we optimized in previous studies (Horáková E, Faktorová D, Kraeva N, et al. Catalase compromises the development of the insect and mammalian stages of Trypanosoma brucei. FEBS J. 2020;287(5):964-977.)

Eukaryotic cell lines

Policy information about cell lines

Cell line source(s)	pleomorphic cell line T. brucei brucei AnTat1.1 90:13 (Engstler et al., Cold shock and regulation of surface protein trafficking convey sensitization to inducers of stage differentiation in Trypanosoma brucei." Genes & development vol. 18,22 (2004): 2798-811.)<br>monomorphic Lister 427 'single-marker' (SM) BF cell line and procyclic 29-13 (Wirtz, E et al. "A tightly regulated inducible expression system for conditional gene knock-outs and dominant-negative genetics in Trypanosoma brucei." Molecular and biochemical parasitology vol. 99,1 (1999): 89-101.)<br>Trypanosoma brucei gambiense LiTat1.3 ELIANE strain and T.b. gambiense strains Bosendja and PA (Fontaine, Frédéric et al. "APOLs with low pH dependence can kill all African trypanosomes." Nature microbiology vol. 2,11 (2017): 1500-1506.)
Authentication	RRID: SCR_004786
Mycoplasma contamination	Cell lines were not tested for Mycoplasma contamination.
Commonly misidentified lines (See ICLAC register)	No commonly misidentified cell lines were used.

Animals and other organisms

Policy information about studies involving animals; ARRIVE guidelines recommended for reporting animal research

Laboratory animals	BALB/c mice, cohort of female or male, 4-6 weeks-old.<br>Mice were maintained at 21 ± 1°C with 55% relative humidity on a 12-h light/12-h dark schedule.<br><br>Glossina morsitans morsitans (1-2 days old) teneral flies from the colony of the Institute of Tropical Medicine (ITM, Antwerp, Belgium) were used. Experimental flies were maintained in standardized environmental conditions of 26°C, 70% relative humidity, and a 12-h light/12-h dark schedule.
Wild animals	The study did not involve wild animals.
Field-collected samples	No field collected samples were used in the study.
Ethics oversight	In Czech Republic, the research was approved by the Central Commission for Animal Welfare, Biology Centre (protocol No. 28/2016). All experimental procedures complied with the Czech law (Act No. 246/1992). In Belgium, the research was approved by the animal ethics committee of the Institute for Molecular Biology and Medicine and the Institute of Tropical Medicine (tsetse fly infection experiment). All mice were housed in a pathogen-free facility and the experiments were performed in compliance with the relevant laws and institutional guidelines (license LA1500474).

Note that full information on the approval of the study protocol must also be provided in the manuscript.
